# Crack Propagation in As-Extruded and Heat-Treated Mg-Dy-Nd-Zn-Zr Alloy Explained by the Effect of LPSO Structures and Their Micro- and Nanohardness

**DOI:** 10.3390/ma14133686

**Published:** 2021-07-01

**Authors:** Petra Maier, Benjamin Clausius, Asta Richter, Benjamin Bittner, Norbert Hort, Roman Menze

**Affiliations:** 1 School of Mechanical Engineering, University of Applied Sciences Stralsund, 18435 Stralsund, Germany; benjamin.clausius@hochschule-stralsund.de; 2 Department Engineering Physics, Technical University of Applied Sciences Wildau, 15745 Wildau, Germany; asta.richter@th-wildau.de; 3 MeKo Laserstrahl-Materialbearbeitungen e.K., 31157 Sarstedt, Germany; bittner@meko.de (B.B.); menze@meko.de (R.M.); 4 Helmholtz-Zentrum Hereon, 21502 Geesthacht, Germany; norbert.hort@hereon.de

**Keywords:** magnesium, Mg-Dy-Nd-Zn-Zr, RESOLOY^®^, LPSO phases, crack propagation, hardness, nanoindentation

## Abstract

The investigation of the crack propagation in as-extruded and heat-treated Mg-Dy-Nd-Zn-Zr alloy with a focus on the interaction of long-period stacking-ordered (LPSO) structures is the aim of this study. Solution heat treatment on a hot extruded Mg-Dy-Nd-Zn-Zr (RESOLOY^®^) was done to change the initial fine-grained microstructure, consisting of grain boundary blocky LPSO and lamellar LPSO structures within the matrix, into coarser grains of less lamellar and blocky LPSO phases. C-ring compression tests in Ringer solution were used to cause a fracture. Crack initiation and propagation is influenced by twin boundaries and LPSO lamellae. The blocky LPSO phases also clearly hinder crack growth, by increasing the energy to pass either through the phase or along its interface. The microstructural features were characterized by micro- and nanohardness as well as the amount and location of LPSO phases in dependence on the heat treatment condition. By applying nanoindentation, blocky LPSO phases show a higher hardness than the grains with or without lamellar LPSO phases and their hardness decreases with heat treatment time. On the other hand, the matrix increases in hardness by solid solution strengthening. The microstructure consisting of a good balance of grain size, matrix and blocky LPSO phases and twins shows the highest fracture energy.

## 1. Introduction

Research on Mg-RE alloys covers many aspects: strength and ductility [1], creep [2], fatigue [3], corrosion [4,5], and their biological performance as biodegradable implants [6,7]. However, there are only a few studies on the fracture mechanics, toughness and the influence of the microstructure on the crack propagation, especially in Mg-RE alloys containing Zn and therefore consisting of long-period stacking-ordered (LPSO) structures. 

Resoloy^®^, an Mg-Dy-Nd-Zn-Zr alloy high in Dy, was developed specifically for absorbable implants by Meko Laser Materials Processing in Sarstedt and the Helmholtz-Zentrum Geesthacht in Germany, is internationally patented [8] and shows excellent strength and ductility. The high elasticity of Resoloy provides excellent fatigue life in air [9,10,11]. Dy, having a high solubility in Mg, can adjust in combination with other alloying elements, such as Gd, Zr, and Nd, both the mechanical and corrosion properties by heat treatment and, according to in vitro studies, Mg-Dy alloys show good cytocompatibility [12,13]. Mg-RE-Zn alloys, where RE are elements such as Y, Gd, Tb, Dy, Ho, Er, and Tm, form novel LPSO structures and show improved strength, fracture toughness, corrosion resistance and fatigue strength and by deformation kinks also remarkable deformability [14]. The LPSO phases arrange periodically in the Mg basal planes, forming a well-ordered structure [15], and appear either block-shaped within the grain and/or at the grain boundaries, or needle-like (lamellar) shaped inside the grains [16]. Studies on cast and extruded Mg-RE-Zn alloys show, in dependence on their initial microstructure, their thermo-mechanical treatment or post-heat treatment changes in the morphology of the LPSO phases. Blocky and lamellar LPSO phases can dissolve into the Mg-matrix and saturated solid solution is formed [17,18], blocky LPSO phases can transform into lamellar LPSO structures [19,20,21,22], the lamellar structure can change its periodical intervals [23] and higher blocky and lamellar LPSO structures can form during ageing treatment [24].

Resoloy^®^, in previous studies, shows a strong grain size increase by solution heat treatment at 500 °C and a change in the amount of blocky and lamellar LPSO structures with increasing solution heat treatment time [25,26]. Lamellar LPSO structures within the matrix, most pronounced in the fine-grained as-extruded microstructure, provide a uniform corrosion at the highest strength and hardness. The coarse-grained microstructure after solution heat treatment revealed large corrosion pits. However, during stress corrosion, no crack initiated from them. The fracture toughness shows the highest value where the microstructure consists of a good balance of moderate grain size, grains saturated by solid solution and an optimized number of blocky and lamellar LPSO structures [26]. Preliminary investigations show that blocky and lamellar LPSO phases hinder crack growth by redirecting the crack along the interface of the blocky LPSO phase and the Mg-matrix, or by redirecting along the orientation of the lamellar LPSO phases, which differs between neighboring grains. Here the crack follows the softer Mg-matrix.

While some microstructural features cause crack initiation under mechanical loading or stress corrosion [27], these features also hinder crack propagation and increase the fracture toughness: low angle grain boundaries [28], twinned grains [29,30], second phases [28], LPSO structures [31,32], and crystallographic planes [33]. Charpy tests [34], slow rate tensile tests [35] or U-bent tests [36] are often used to determine the fracture toughness under stress corrosion. This study applies a C-ring compression test to investigate the fracture toughness and crack propagation. Similar tests have already been done on the Mg-Dy-Nd alloy [37]. Dislocations in twinned regions or grains with lamellae LPSO structures will have short distances to move and will therefore pile up at these obstacles and can lead to crack initiation and reduced toughness [38]. The stress pile-up will depend on the coherence of the slip planes. The interfaces between LPSO structures and the Mg-matrix are preferential sites for micro-crack initiation: a blocky LPSO structure does not match the deformation of the Mg-matrix and therefore stress accumulates at the interface. The merging of cracks in adjusted grains resulted in crack propagation. Cracks can pass through grain boundary (gb) LPSO phases into the adjacent grain. The generation of cavities and crack blunting was observed when the cracks propagation direction deviated remarkably from the orientation of the LPSO slip bands [32].

To evaluate the hardness of individual microstructure features, tensile and compression tests and hardness measurements of higher loads reaching their limits were performed. Nanoindentation measurements with a small indent size show the higher hardness of precipitates in Mg alloys [39], the influence of solid solution strengthening on the nanohardness in Mg-Ga alloys [40], the influence of the temperature on thermo-mechanical processing in Mg-Li-Al [41] and the transition of elastic to elastic/plastic deformation [42]. Crack initiation by second phases [43] or other microstructural features refer back to their brittleness and high hardness. Research on Mg-Zn-Y-Mn shows higher nanohardness (at an applied load of 5 mN) of the blocky LPSO phases compared to the Mg-matrix, which will explain their brittleness [44]. Individual microhardness measurements (applied load 100 N) on Mg-Gd-Y-Zn-Zr [45] also show a higher hardness of blocky LPSO phases. Nanohardness measurements on ZE41 doped with Ce show, in dependence on the Ce-content, the indentation size effect (the hardness value decreases with the increasing indentation test load) and also the reverse indentation size effect due to microstructure modification [46].

The motivation of this study is to explain the crack propagation in different microstructures by their microstructural features, especially by lamellar and blocky LPSO structures and their micro- and nanohardness. With this knowledge, the microstructure can be optimized and mechanical properties can be tailored to fit their application.

## 2. Materials and Methods

The Mg-Dy-Nd-Zn-Zr alloy was cast using permanent mould direct chill casting [47] at the Helmholtz-Zentrum in Geesthacht, Germany. After a short annealing at 500 °C for 15 min, tubes were indirectly hot-extruded at the Extrusion Research and Development Center TU Berlin at an overall temperature of 400 °C, a ram speed of 1.5 mm/s and an extrusion ratio of 19:1. The as-extruded tubes had an outer diameter of 35 mm with a wall thickness of 5 mm. The chemical composition of Dy and Zn were analyzed by using the X-ray micro fluorescence M4 Tornado (Bruker, Billerica, MA, USA), and Nd and Zr by using a spark optical emission spectroscopy Spectrolab M12 Hybrid (Ametek-Spectro, Kleve, Germany). Annealing and solution heat treatment were performed at two temperatures: 200 °C for 4 h and 500 °C for 24 h, 48 h, and 72 h. The samples were cooled down in warm water at a temperature of 55 °C. The rings of the tubes for metallographic and hardness investigations were prepared for optical microscopy by grinding with SiC paper to a grit size of 4000. Afterwards, samples were polished with 3 and 1 µm water-free diamond paste followed by 0.04 μm OPS colloidal silica. The samples were then cleaned with ethanol and dried with hot air. The samples were etched in a solution prepared with 4.2 g picric acid, 10 mL acetic acid 100%, 10 mL distilled water, and 70 mL ethanol. Micrographs were obtained on a Leica DMi8 A optical microscope (Leica Microsystems GmbH, Wetzlar, Germany). IMAGIC IMS software was used for the evaluation of the micrographs, including grain size measurements using the line intercept technique. The optical micrographs were used to characterize microstructural features, such as the location and the amount of the LPSO phases. 

C-ring samples with a width of 10 mm were machined from the extruded tubes with an outer diameter of 34 mm and a wall thickness of 2 mm. Their position in the initial extruded tubes can be seen in [37]. C-ring samples were compressed at 2 mm/min in a testing machine up to fracture in Ringer solution at 37 °C. The study on Mg-Dy-Nd [37] provides detailed information on the C-ring compression test in Ringer and the study in [26] presents data from the force–displacement curves, such as force and displacement at crack initiation and its fracture toughness. The force-displacement curves from the C-ring compression tests were used to discuss the fracture behavior [26], mostly by using the fracture energy for fracture toughness, evaluated by the area underneath the force-displacement curve during crack propagation. Because of the test end criterion of falling below a force of 5 N during the C-ring compression test, the samples did not fail completely and, as well as the crack flanks, the crack tip could also be investigated. Cross-sectional micrographs of the fractured C-rings are used to evaluate the influence of the microstructural features such as twins and LPSO phases, either as lamellar matrix structures or as blocky matrix- or gb- phases, on the crack propagation.

The Vickers hardness HV1 and HV0.2 were tested with a universal hardness testing machine ZHU2.5 by Zwick (ZwickRoell, Ulm, Germany), with up to 30 indents per condition applying HV1 and up to 15 indents using HV0.2. Individual values for HV0.005 were tested with a Zeiss Neophot 2 (Carl Zeiss Jena GmbH, Jena, Germany). In addition to the mean values, minimum and maximum values are reported and used for discussion. Due to the small indent size, only the microhardness by Vickers by HV0.005 could be used to determine the hardness of the blocky LPSO phases. Nanoindentation measurements were performed using the electrostatic transducer of the UBI 1 Hysitron TriboScope (Hysitron, Inc., Minneapolis, MN, USA) with a diamond 90° cube corner tip. The detailed methodology is described in [39,48]. Progressive multi-cycles in force control applying 12 consecutive loading–unloading cycles with the load being increased to 2 mN were performed to also measure the load-dependent hardness. The mean value m_i_ of each individual multi-indent was calculated by the hardness values in the range of the lowest dependence on the load applied, and its standard deviation was kept below 0.1 GPa. Their mean, minimum and maximum values are presented. 

## 3. Results

### 3.1. Characterization of the Microstructure

**Figure 1:**
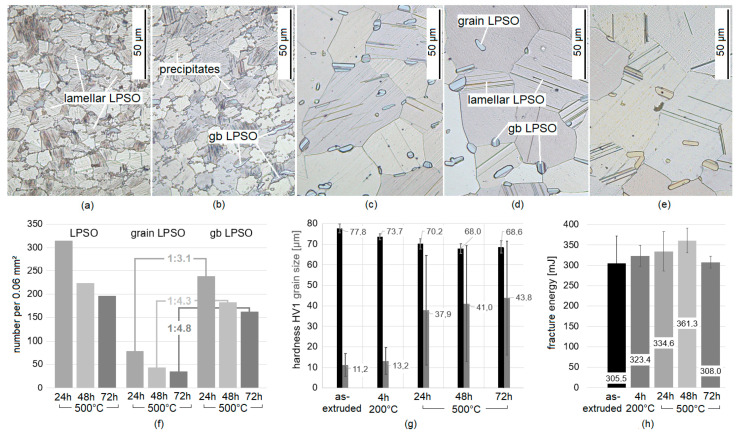
Microstructure of Mg-Dy-Nd-Zn-Zr alloy in as-extruded and after 200 °C for 4 h (**a**,**b**), showing LPSO lamellae within the grains, precipitates and grain boundary (gb) long-period stacking-ordered (LPSO), and after 500 °C for 24 h, 48 h, and 72 h (**c**–**e**), showing LPSO lamellae within Mg-matrix and blocky LPSO within the grain and at the grain boundaries, their amount within a certain area (**f**), its grain size and hardness HV1 (**g**) and its fracture toughness, based on [26] (**h**).

The Mg-Dy-Nd-Zn-Zr alloy consists of 12.63 wt.% Dy, 1.05 wt.% Nd, 0.94 wt.% Zn and 0.075 wt.% Zr. Fe, Cu and Ni do not exceed 0.001 wt.%. Figure 1a–e show representative microstructures of the fine-grained extruded (Figure 1a) and heat-treated condition of 200 °C for 4 h (Figure 1b) and coarse-grained solution heat-treated conditions of 500 °C for 24 h (Figure 1c), for 48 h (Figure 1d) and for 72 h (Figure 1e). In the fine-grained microstructure, all grains contain LPSO lamellae (thin black lines) in narrow distance and blocky LPSO at the grain boundaries and precipitates are also found (Figure 1a,b). Micrographs of coarse-grained heat-treated condition reveals microstructural features such as the lamellar LPSO structures with the Mg-matrix and the blocky LPSO phases within the grains and at the grain boundaries, see highlighted in Figure 1d. As seen in Figure 1f, the number of blocky LPSO phases decreases over the solution heat treatment time, within the grain as well as the gb. The reduction towards 48 h is higher than towards 72 h. There are significantly more gb blocky LPSO than blocky LPSO phases within the grain. The blocky LPSO phases at the grain boundaries are more resistant to dissolution during heat treatment; see the relation values in Figure 1f, which are based on the same area of the microstructure. After the solution heat treatment at 500 °C for 24 h there are ~3 times as many gb blocky LPSO phases in relation to the inner grain ones, and for 72 h already ~5 times as many. Without treating the blocky LPSO phases as grains, the grain size increases by solution heat treatment at 500 °C with significant scattering, and the hardness HV1 decreases (with the lowest mean value at 48 h); see Figure 1g.

Whereas the fine-grained microstructure shows the highest number of grains at grain sizes around 10 µm and with no grains larger than 30 µm, the coarse-grained microstructure shows the highest number of grains around 24 µm at 500 °C for 24 h, at around 28 µm for 48 h and around 34 µm for 72 h. The blocky LPSO phases can reach a size of 10 µm; however, there are also phases in the sub-µm range. According to the mean value, the hardness HV1 decreases slightly by the heat treatment of 200 °C for 4 h (acting similar to stress relief), and further by solution heat treatment of 500 °C for 48 h, but increases slightly towards the 72 h heat treatment time (Figure 1g). The fracture toughness shows its highest value at the solution heat treatment at 500 °C for 48 h; see Figure 1h.

### 3.2. Crack Propagation

**Figure 2:**
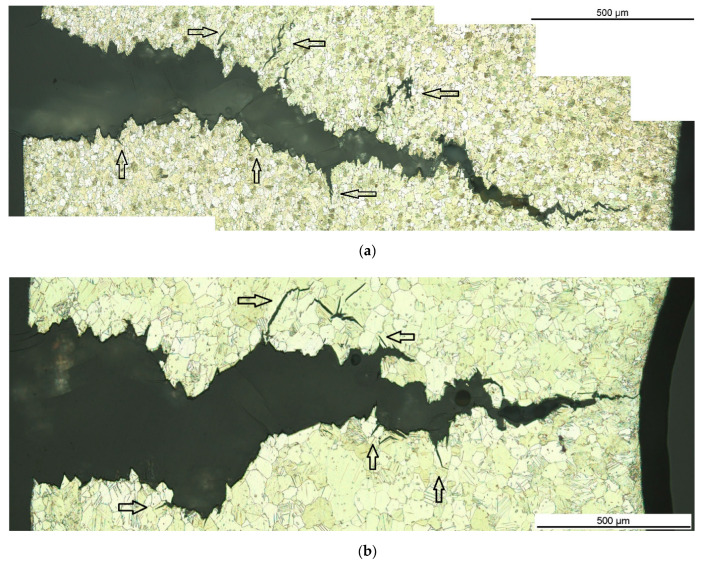
Representative cross-sectional optical micrographs of the crack path in the Mg-Dy-Nd-Zn-Zr alloy C-ring samples for the fine-grained microstructure: heat-treated at 200 °C for 4 h (**a**) and for the coarser-grained microstructure: solution heat-treated at 500 °C for 24 h (**b**).

The C-ring compression tests were stopped when the significant force reduction during crack growth was exceeded (see force-displacement curves in [26]), so the samples did not fracture completely into two parts and the tip could be investigated. Figure 2 shows representative cross-sectional micrographs of the crack path in the C-ring samples for both the fine-grained microstructure by the example of the heat-treated condition at 200 °C for 4 h (Figure 2a) and the coarser-grained condition with the solution heat-treated condition at 500 °C for 24 h (Figure 2b). The cross-sections show sub-cracks (see arrows) of the main cracks, mainly when the initial neutral phase of the C-ring sample is past. From the cross-sectional micrographs in Figure 2b it can be stated that the fracture shows transgranular cracking. Furthermore, it can be seen that the grains surrounding the crack path in the coarser-grained microstructure are twinned, being more pronounced at the compression side around the crack tip. Before the crack has reached the compressed side of the C-ring sample, the twins have formed, and therefore play a role in further crack growth.

**Figure 3:**
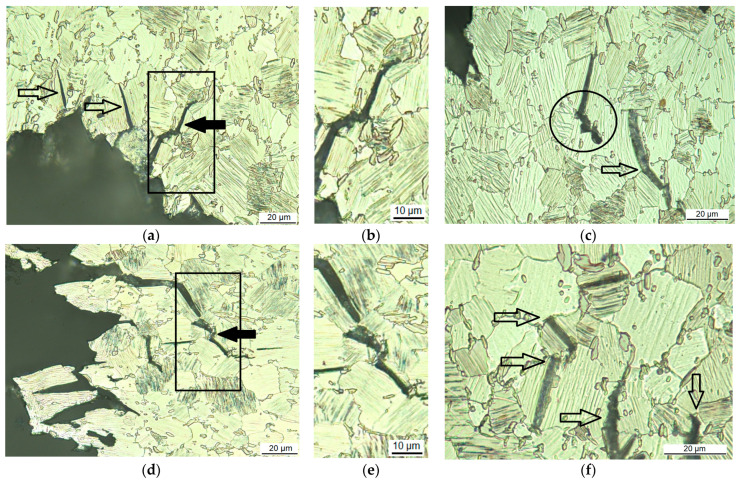
Cross-sectional optical micrographs of the crack path in the Mg-Dy-Nd-Zn-Zr alloy C-ring samples for the fine-grained microstructure: extruded (**a**–**c**) and heat-treated at 200 °C for 4 h (**d**–**f**).

Micrographs with higher magnifications present the interaction of the propagation of the sub-cracks branching off the main crack with the microstructural features. LPSO lamellae appear as thin, sometimes single lines, within a single grain in parallel formation and stops and starts at different locations. The contrast in between these lamellae does not differ from the rest of the grain. Twin boundaries show as thin parallel lines with a different contrast in between compared to the contrast outside the twin. The twins start at gb or other interfaces and run to the next interface or twin boundaries run towards each other within the grain, forming elliptical shapes. Figure 3 shows the cross-sectional micrographs of the crack path in the fine-grained microstructure, in Figure 3a–c of the as-extruded condition and in Figure 3d–f when heat-treated at 200 °C for 4 h, in which the grains have grown slightly with little reduction of the hardness. It can be clearly seen that the sub-cracks follow the direction of the LPSO lamellae of the individual grain (see arrows in Figure 3a and in the magnified area in Figure 3b (highlighted by right black arrow in Figure 3a) and are sidetracked by blocky LPSO phases, precipitates or by grain boundaries (see circled void area and the arrow for the gb in Figure 3c). Weak interfaces between Mg-matrix and precipitates or blocky LPSO phases cause nucleation of voids under loading, followed by void coalescence, which supports crack growth. Some blocky LPSO phases at the grain boundaries (see black arrow in Figure 3d and its magnified area in Figure 3e) form a gb network, bridging the micro-cracks via the interface from grain to grain, where it then propagates in the direction of the LPSO lamellae. The gb is certainly an obstacle, which leads to crack blunting (see top two arrows in Figure 3f), especially when there is no direct new slip plane in the adjacent grain. Here the blocky LPSO phases at the gb carry the crack by a few µm along it. The blocky LPSO phases within the grains also hinder crack growth, and the straight line of the crack opening is redirected (see two bottom arrows in Figure 3f indicating a redirected crack and blocky LPSO near the crack tip). Intergranular cracking cannot be seen, and extensive twinning has also not been found, most likely due to the high amount of LPSO lamellae.

**Figure 4:**
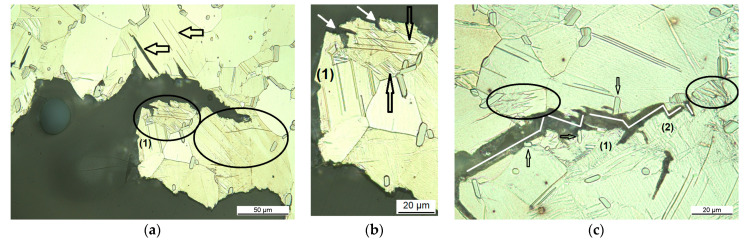
Cross-sectional optical micrographs of the crack path in the Mg-Dy-Nd-Zn-Zr alloy C-ring samples for the coarse-grained microstructure: solution heat-treated at 500 °C for 24 h (**a**,**c**), (**b**) magnified area from micrograph (**a**) circle (1).

By examining the microstructure along the crack path of the solution heat-treated conditions at 500 °C, where the grains have grown, it can be seen that the grains are twinned and the amount of twins increases towards the compression side of the C-ring sample. During C-ring deformation the crack opens at the tensile side while the grains at the compression side are exposed to larger strain before the crack “arrives” and twins to a larger extent. Figure 4 shows cross-sectional micrographs of the crack path in the coarse-grained microstructure, the solution heat-treated condition at 500 °C for 24 h. That the crack propagates parallel to the lamellar LPSO phases can be seen in Figure 4a; see arrows highlighting the crack parallel to the LPSO lamellae. Some micro-cracks do not seem to be connected to the main crack; see top left. However, the micrographs show only one individual cross-section; further into or out of the visible plane, as these cracks might be connected to the main crack. The two grains below the crack tip in Figure 4a show large twins, which also clearly interfere with the crack propagation, as the twins redirect the crack as it grows, and the crack surface appears jagged and uneven (circled areas). The circled area labeled with number (1) in Figure 4a is magnified in Figure 4b, where the black arrows highlight some twins and the white arrows point out sub-cracks parallel to the twins. LPSO lamellae in this grain are appearing almost horizontally, at 45° to the twins. Figure 4c shows the same appearance of jagged and uneven crack flanks (white line within the crack). Here it can be seen that the crack interferes the twin boundaries; twinned grains around the crack are circled and labelled numbers (1) and (2). The blocky LPSO are passed sideways by the interface (see arrows pointing out two of the blocky LPSO phases).

**Figure 5:**
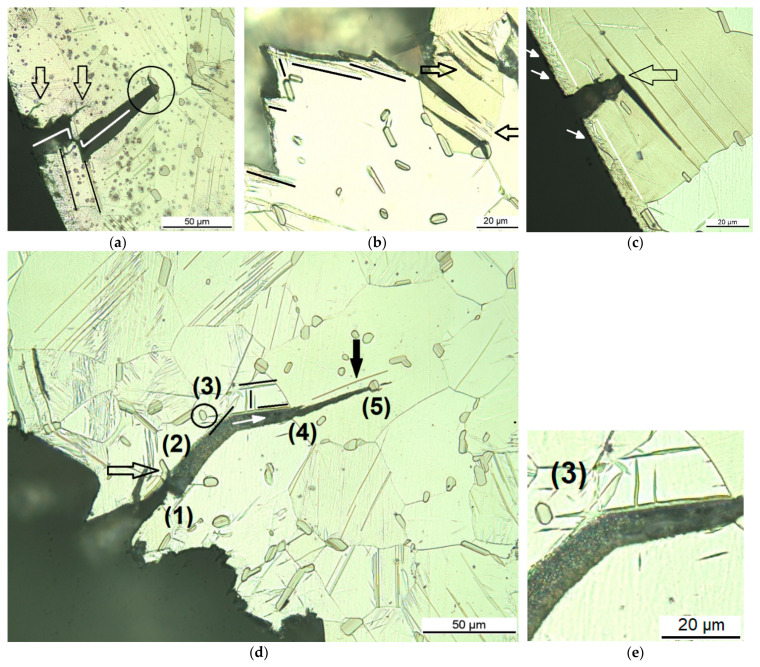
Cross-sectional optical micrographs of the crack path in the Mg-Dy-Nd-Zn-Zr alloy C-ring samples for the coarse-grained microstructure: solution heat-treated at 500 °C for 48 h (**a**–**d**), (**e**) magnified area from micrograph (**d**).

Figure 5 shows the crack path in the solution heat-treated condition at 500 °C for 48 h. The micrographs show very clearly the interaction of the microstructural features with the crack propagation. In Figure 5a the cracks stop in a blocky LPSO phase, which has the lamellar structure in a 90-degree angle to the crack propagation. The crack has blunted and the interface between the LPSO phase and the Mg-matrix voids will have supported the propagation of the crack (see circle). The crack shown in Figure 5a is a very short surface crack, which shows some even shorter sub-cracking (arrows), here most likely caused by hydrogen embrittlement, as the C-ring samples have been compressed in the Ringer solution. However, a redirection by a LPSO lamella can be seen (black lines), and the further crack opening caused the zig-zag (white line within the crack). The sub-cracks in the micrograph in Figure 5b run along the matrix LPSO lamellae (top arrow), and twin boundaries are also formed, also supporting crack growth (bottom arrow). The bottom sub-crack is connected to a blocky LPSO phase, but it is not clear where this crack initiated. The sub-crack could also be connected with the main crack in another plane. The orientation of the blocky LPSO phase and the crack within the grain are not too far off to cause the crack to propagate via the interface. The neighboring grains do not show any twin boundaries and matrix LPSO lamellae and do not show micro-cracks. However, cracked blocky LPSO phases have been found to initiate cracks. The black lines in the brighter, large grain in Figure 5b are parallel to twin boundaries. Figure 5c shows clearly that a crack gets redirected by matrix LPSO lamella after blunting when the crack growth direction is 90-degree off the LPSO lamella (arrow). The micro-crack opens by propagating towards the gb. The crack might be initiated by the twin boundaries (white arrows indicate their direction), which are seen at the surface reaching a few µm (white line) and will be a result of tensile stress during loading. The sub-crack in Figure 5d branches off a matrix LPSO lamella (1), bypasses a blocky LPSO phase, which is pointed out by the arrow (2) and is tracked by a twin boundary (twins are highlighted by black parallel lines near (3), circle indicates the end of the twinned area very close to a blocky LPSO). Figure 5e shows the magnified twinned area near (3). Then the crack is redirected to the right by another twin boundary, see white arrow, which crosses to the adjusting grain (4) by either a matrix LPSO lamellae, being parallel to the one highlighted by the black arrow pointing downwards, by a twin boundary or by a slip plane and propagates via the interface of a blocky LPSO phase, which cannot be crossed (5), because the orientation is 90-degrees off the crack growth direction. 

**Figure 6:**
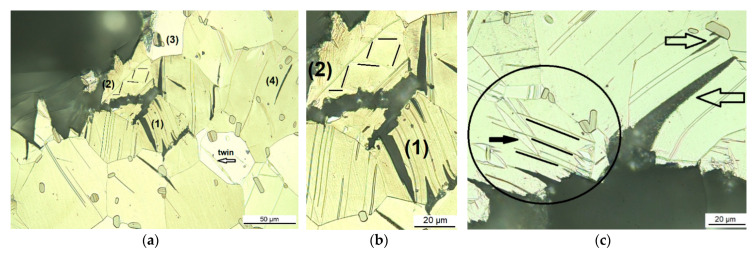
Cross-sectional optical micrographs of the crack path in the Mg-Dy-Nd-Zn-Zr alloy C-ring samples for the coarse-grained microstructure: solution heat-treated at 500 °C for 72 h (**a**,**c**), (**b**) magnified area from micrograph (**a**).

The cross-sectional micrographs in Figure 6 show the crack path in the solution heat-treated condition at 500 °C for 72 h. Figure 6a,b show a region of a few micro-cracks, either along the lamellar matrix LPSO structures (1) or along twin boundaries (2), where the black lines point out the twins. Additionally, a weak interface between the Mg-matrix and a blocky LPSO phase forming a void can be seen in Figure 6a at the top middle (3) as well as a sub-crack parallel to LPSO lamellae (4). When there are coarser grains free of twins and blocky LPSO phases, as in Figure 6c, the crack runs continuously, following the lamellar matrix LPSO structure through the grain (bottom arrow), and is deflected at the approaching gb or rather by the different orientation of the adjacent grain or its microstructural features. Above the larger crack, a micro-crack can be seen along an LPSO lamella, near the blocky LPSO (top arrow).

The circle in Figure 6c highlights an area, where twins and LPSO lamellae can be very well distinguished: a large twin forming the elliptical shape with a lighter contrast in between the twin boundaries is pointed out by the black arrow, whereas LPSO lamellae appear as thin, parallel lines (see three lines added parallel to them). 

### 3.3. Microhardness HV0.2

**Figure 7:**
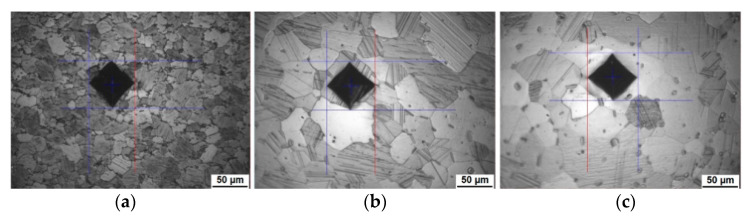
Optical micrographs of Mg-Dy-Nd-Zn-Zr alloy with HV0.2 indents in the: as-extruded condition (**a**) and solution heat-treated condition at 500 °C for 48 h (**b**,**c**), grain with matrix LPSO lamellae (**b**) and grain without matrix LPSO lamellae (**c**).

To characterize the microstructural features regarding their hardness, individual regions were tested with the Vickers hardness HV0.2. The Vickers hardness indents of HV1 of this Mg-Dy-Nd-Zn-Zr alloy (Figure 1c) have diagonal length values of around 160 µm, which are much larger than the average grain size, of both the fine- and coarse-grained microstructure; see also Figure 1c. The HV0.2 indents with diagonal lengths of around 70 µm are still too large for the fine-grained microstructure; see here Figure 7a for the as-extruded condition, where the indent covers several grains and LPSO phases. According to, for example, the grain size of the solution heat-treated condition at 500 °C at 48 h with 41.0 µm ± 28.2 µm, there are large grains in the coarser-grained microstructure, which are able to be tested individually. Figure 7b,c show indents in these large grains, in a grain with matrix LPSO lamellae (b) and in a grain without matrix LPSO lamellae (c). With care, grains large enough as well as grains without blocky LPSO have been tested. However, the indents are still far too large to test individual blocky LPSO phases, having sizes up to 10 µm, although most of them are in a range of a few µm.

**Figure 8:**
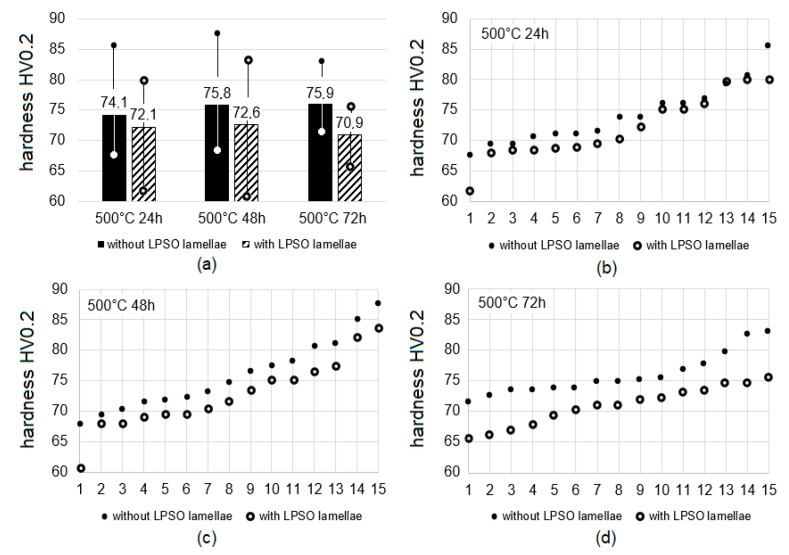
Hardness HV0.2 of grains with and without matrix LPSO lamellae influence by solution heat treatment time at 500 °C: mean values with minimum and maximum value (**a**), values sorted according to their size at 24 h (**b**), 48 h (**c**) and 72 h (**d**).

Figure 8a shows the mean values and the minimum and maximum values of the grains with and without lamellar LPSO within the Mg-matrix in the solution heat-treated conditions. The grains without lamellar LPSO structures are harder than the grains with LPSO lamellae, although there is a high scattering (difference between the minimum and maximum value). The difference in hardness increases with the increasing solution heat treatment time. It is noticeable that the scattering in the hardness values is the highest for the solution heat-treated condition at 500 °C for 48 h. There is a slight increase in the hardness of the grains without lamellar LPSO structures with the solution heat treatment time, whereas the hardness of the grains with LPSO lamellae shows the lowest hardness value in the solution heat-treated condition at 500 °C for 72 h.

The graphs in Figure 8b–d show the 15 individual values, which provide the mean, minimum, and maximum values presented in Figure 8a. The values are sorted according to their size. The hardness values of the solution heat-treated condition at 500 °C for 24 h are close to each other; for 48 h the difference becomes much clearer, and for 72 h there is no doubt that the grains without lamellar LPSO structures are harder than the ones with LPSO lamellae. For the 72 h heat treatment time, the smallest value for grains without lamellar LPSO structures is higher than the mean values of the grains with LPSO lamellae. Here there are no extreme minimum and maximum values, and this condition seems more homogeneous.

It is worth mentioning that there is no obvious correlation of the number and orientation of LPSO lamellae, or of their spacing in between and the hardness value. The shape of some indents is not exactly symmetrical, but to determine any influence of the direction of the LPSO lamellae, further and detailed investigations are needed.

### 3.4. Microhardness HV0.005

**Figure 9:**
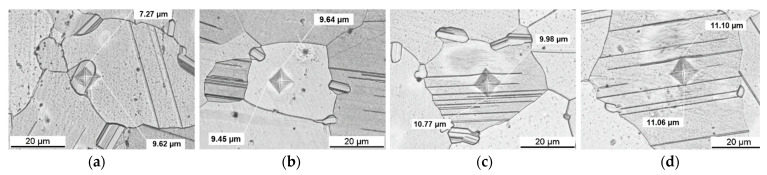
Optical micrographs of Mg-Dy-Nd-Zn-Zr alloy in the solution heat-treated condition at 500 °C for 48 h with HV0.005 indents in: blocky LPSO (**a**), grain without matrix LPSO lamellae (**b**), grain with matrix LPSO lamellae, where lamellae are indented (**c**) and grain with matrix LPSO lamellae, where the indent sits in between the lamellae (**d**).

As seen with the HV0.2 indents (see Section 3.3.), they are too large to test the blocky LPSO phases individually. To reduce the load even further from Vickers HV1 to HV0.2, Vickers HV0.005 has been applied. Figure 9 shows representative optical micrographs of indents in the solution heat-treated condition at 500 °C for 48 h: in a blocky LPSO (Figure 9a), in a grain without matrix LPSO lamellae (Figure 9b), in a grain with matrix LPSO lamellae, where the lamellar region is indented (Figure 9c) and in a grain with matrix LPSO lamellae, where the indent sits in between the lamellae (Figure 9d). The diagonal length values are labeled. With the Vickers hardness HV0.005 indents, some large blocky phases can be indented individually. However, not all indents are used for the calculation of the mean value due to a too strong interference of the interface or the neighboring grain. In this case, the indents are non-square and enlarge with one diagonal length, as seen in Figure 9a. Figure 9b shows that also smaller grains can be tested with this small load. Figure 9c,d show that the indent is deforming some LPSO lamellae. To determine the influence of the number of LPSO lamellae indented, measurements with a higher statistic would be needed.

**Figure 10:**
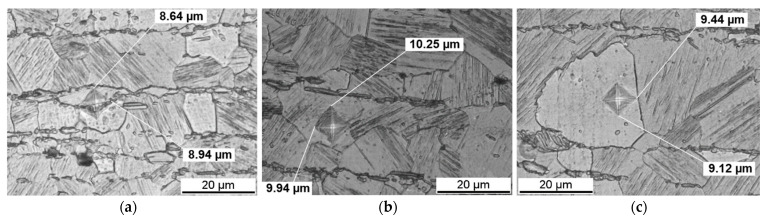
Optical micrographs of Mg-Dy-Nd-Zn-Zr alloy in the as-extruded condition with HV0.005 indents in: blocky LPSO (**a**), grain with matrix LPSO lamellae (**b**), and grain without matrix LPSO lamellae (**c**).

Figure 10 shows optical micrographs of indents in the as-extruded condition, in a blocky LPSO (Figure 10a), in a grain with matrix LPSO lamellae (Figure 10b) and in a grain without matrix LPSO lamellae (Figure 10c). In the as-extruded condition, it is very difficult to test the blocky LPSO on its own, as they are not large enough. Values are presented, but should be treated with some skepticism.

**Figure 11:**
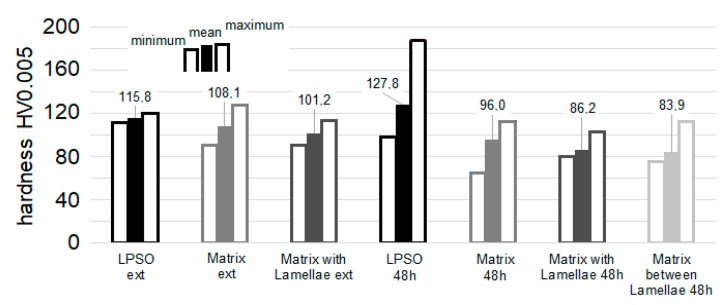
Minimum, mean and maximum values of the hardness HV0.005 in Mg-Dy-Nd-Zn-Zr alloy in the as-extruded and the solution heat-treated conditions at 500 °C for 48 h: in the blocky LPSO phases, in the matrix without and with LPSO lamellae and for 48 h in the matrix in between lamellae a wider distance apart.

The bar chart in Figure 11 shows the mean and minimum and maximum HV0.005 hardness values of the as-extruded and the solution heat-treated conditions at 500 °C for 48 h of the blocky LPSO phases, in the matrix without and with LPSO lamellae and for 48 h in the matrix in between lamellae a wider distance apart. The blocky LPSO phases are harder than all the other regions. Because it is not possible to eliminate the influence of the interface or the neighboring grains of the smaller blocky LPSO phases in the as-extruded condition, it should not be concluded straight away that they are less hard than the blocky LPSO phases in the solution heat-treated conditions at 500 °C for 48 h. The grains of the matrix without lamellar LPSO structures are harder than the grains of the matrix with lamellar LPSO structures, which agrees with the Vickers hardness HV0.2 measurements.

Since the imprints are of a size of around 10 µm, the Mg-matrix in between the LPSO lamellae could be measured individually; its mean value is smaller than the grains of the Mg-matrix with or without LPSO lamellae. Even so, its scattering is rather high. However, the scattering (the difference between the minimum and maximum value) of the hardness value of the blocky LPSO phases is very high (Δ hardness of 90 HV0.005), followed by the Mg-matrix without LPSO lamellae (Δ hardness of 48 HV0.005).

### 3.5. Nanohardness with Multi-Cycle Indentation

**Figure 12:**
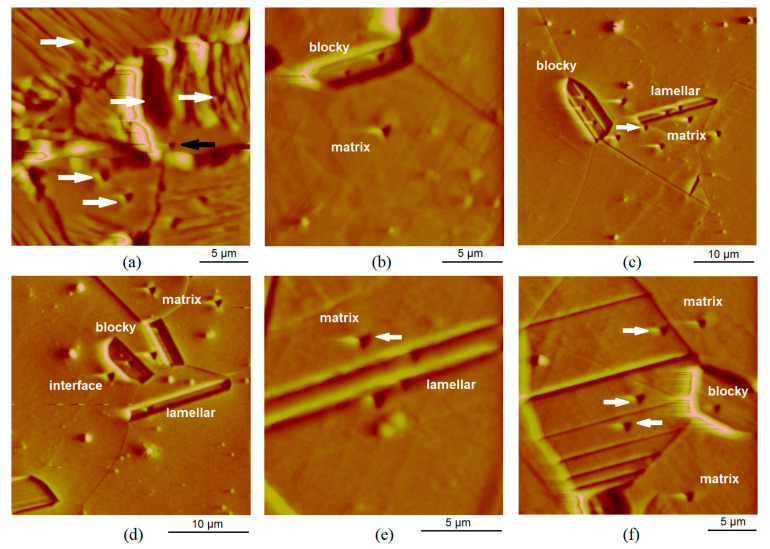
Nanoindenter AFM images with indents of Mg-Dy-Nd-Zn-Zr alloy in as-extruded (**a**) and solution heat-treated at 500 °C: for 24 h (**b**), for 48 h (**c**,**d**) and 72 h (**e**,**f**).

Figure 12 shows atomic force microscopy (AFM) images of Mg-Dy-Nd-Zn-Zr in the as-extruded and solution heat-treated conditions; regions where nanoindents have already been set and can be seen as small triangular-shaped imprints and their small surrounding pile-up areas. It can be seen that the size of the imprints allows the testing of individual microstructural features. The lamellar matrix LPSO structure in the as-extruded condition can easily be seen in Figure 12a. Blocky LPSO phases are more difficult to see and can finally be identified by their hardness values. These blocky LPSO phases appear as networks at the grain boundaries, as evaluated by the light microscopy images. Nanoindents in the blocky LPSO phases can be better seen in the coarser-grained microstructure, as seen in Figure 12b, where two imprints are seen in the phase and one in the matrix of the solution heat-treated condition at 500 °C for 24 h. Blocky and lamellar LPSO structures can be seen in Figure 12c–f. The lamellar structure within the blocky LPSO phases appears in Figure 12c in the solution heat-treated condition at 500 °C for 48 h. LPSO lamellae within the Mg-matrix can be seen in Figure 12e,f (500 °C for 72 h). Besides showing indents in the lamellar LPSO structures with narrow distances (Figure 12e), it also indents in the more widely spaced lamellar LPSO structures in the solution heat-treated condition at 500 °C for 72 h and can be seen in the three imprints in Figure 12f. The spacing of the LPSO lamellae is far enough to measure their hardness independently. Of the other three imprints, two are set in the Mg-matrix and one in a blocky LPSO phase.

**Figure 13:**
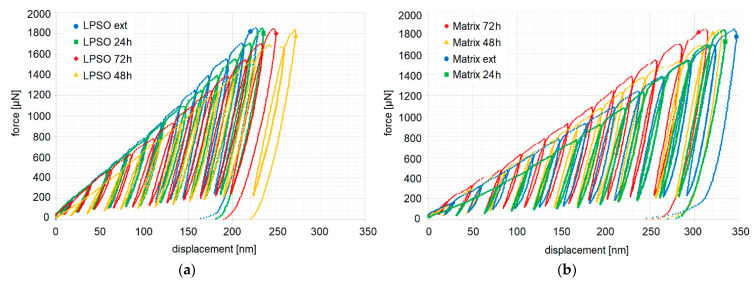
Representative force-displacement curves of multi-cycle indents up to 2 mN in Mg-Dy-Nd-Zn-Zr alloy in the as-extruded and the solution heat-treated conditions at 500 °C for 24 h, 48 h and 72 h: in the blocky LPSO phases (**a**) and in the matrix without LPSO lamellae (**b**).

**Figure 14:**
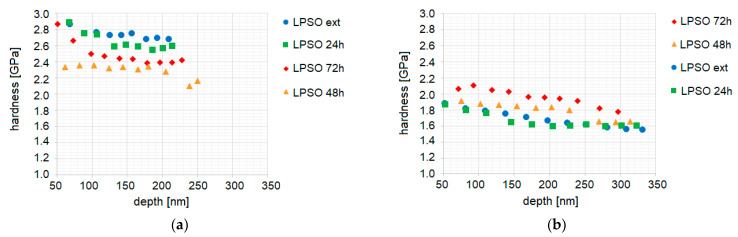
Hardness of the multi-cycle indents up to 2 mN in Mg-Dy-Nd-Zn-Zr alloy in the as-extruded and the solution heat-treated conditions at 500 °C for 24 h, 48 h, and 72 h: in the blocky LPSO phases (**a**) and in the matrix without LPSO lamellae (**b**).

Representative force-displacement curves of indents in blocky LPSO and of the Mg-matrix in Mg-Dy-Nd-Zn-Zr, in both the as-extruded condition and the solution heat-treated conditions at 500 °C for 24 h, 48 h, and 72 h, are shown in Figure 13. The difference in hardness is clearly seen by the dissimilarity of the curves relating to each individual displacement per cycle, as well as the displacement of the 12th and final unloading. That counts for both the blocky LPSO (Figure 13a) and the Mg-matrix (Figure 13b) in dependence on their heat treatment stage, as well as for the blocky LPSO phases (the highest displacement for LPSO phase in the solution heat-treated condition at 500 °C for 48 h is 270 nm) compared to the Mg-matrix, reaching 350 nm in displacement for the as-extruded condition. By looking at the course of the curves of the as-extruded and the solution heat-treated condition at 500 °C for 24 h, it becomes apparent that, up to a displacement of 250 nm, the as-extruded condition shows higher hardness values, but above 250 nm the picture changes (its hysteresis loop moves over to the right). That the hardness values are influenced by the load applied is better seen in Figure 14, where the hardness values are shown in dependence on the indent depth. The values shown in Figure 14 agree with the force-displacement curves in Figure 13. To avoid surface effects and guarantee real values, the x-axes start at 50 nm. It can be seen in Figure 13a that the blocky LPSO phases show the least load-dependence within the range 100–225 nm. The hardness values of small indentation depths are in general higher than those of deeper indents. The hardness values of the LPSO phase in the solution heat-treated condition at 500 °C for 48 h at the depths of 240 nm and 250 nm are noticeably smaller than the less deep ones up to 210 nm (Figure 14b).

The depth-dependent hardness of the blocky LPSO phases has the highest value in the as-extruded material (Figure 14a). With heat treatment time the hardness of the blocky LPSO phases decreases. However, this decrease is not proportional to the heat time. The LPSO phases in the material after heat treatment of 48h show the lowest hardness values of the investigated LPSO structures. Figure 14b show that the depth-dependent hardness of the matrix has the highest value in the heat-treated material of 72 h, followed by heat treatment of 48 h and as-extruded and heat treatment of 24 h.

The load-displacement curves of the solution heat-treated condition at 500 °C for 48 h in Figure 13a show a pop-in between cycles 10 and 11. This abrupt change in displacement is mostly caused by strain bursts; however, these do not occur very often in this alloy. The last two cycles 11 and 12 stand out clearly. According to Figure 14b, the hardness values of the Mg-matrix decrease with the increasing load. The matrix of the solution heat-treated condition at 500 °C for 24 h seems to have the most stable nanomechanical properties when a displacement of 150 nm is exceeded.

**Figure 15:**
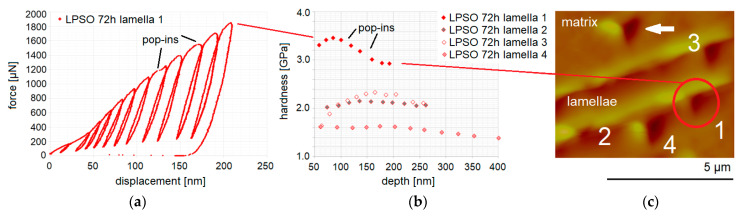
Lamellar LPSO structured region of narrow distance within Mg-matrix after heat treatment of 72 h: force–displacement curve of multi-cycle indent up to 2 mN of LPSO lamella 1 (**a**), its hardness over depth curve with further lamellae 2 to 4 (**b**) and their nanoindenter AFM image, enlarged from Figure 12e (**c**).

The highest dependence of the hardness on the load applied has been found for the regions of narrow lamellar LPSO structures within the Mg-matrix, as seen in Figure 15 for the heat-treated condition at 500 °C for 72 h as a representative example. The variation in distances between each individual cycle regarding their displacements (Figure 15a for indentation 1 in Figure 15c, which is an enlarged image from Figure 12e with one more indent with number 2) leads to the hardness values presented in Figure 15b (top range). Starting with two small hardness values and followed by higher hardness values (not shown in Figure 15b, because the hardness values presented start at a depth of reaching 50 nm), the hardness increases up to a dominant surface maximum 3.46 GPa at 84 nm and then decreases down to 2.93 GPa at 193 nm. The two pop-ins, at 1200 µN at 126 nm, causing a wider space between cycles 7 and 8, and at 1540 µN at 170 nm, causing a wider space between cycles 9 and 10, mark the breakthrough of the harder LPSO lamella or slipping of the tip into the softer surrounding area. The decrease in hardness has stopped for cycles 11 and 12. Where the imprints finally sit is seen in Figure 14 (red cycle labelled with 1). In addition, the indentations 2 and 3 show a strong dependence of the hardness on the load applied (see Figure 15b). The multi-cycle indent 4 appears rather stable; its imprint is in the interface of the Mg-matrix and the LPSO lamella and shows a low overall hardness. The imprint, which is marked in Figure 15c with the white arrow is set in the Mg-matrix, and its multi-cycle hardness values are not presented in the graph in Figure 15b, but the hardness values would be hardness above values of the interface indent 4, starting just above 2 GPa with the final hardness value of 1.6 GPa at 330 nm. With that, the interface hardness shows the lowest values.

**Figure 16:**
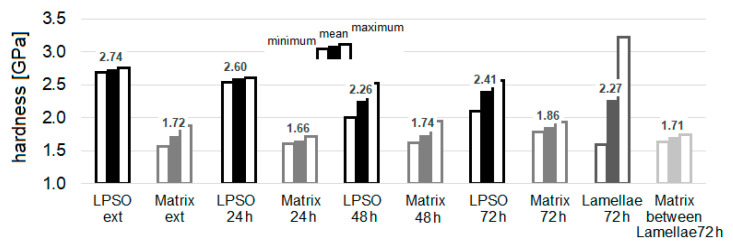
Minimum, mean, and maximum values of the hardness of the multi-cycle indents in Mg-Dy-Nd-Zn-Zr alloy, in the range 100–300 nm in depth, in the as-extruded and the solution heat-treated conditions at 500 °C for 24 h, 48 h, and 72 h: in the blocky LPSO phases, in the matrix without LPSO lamellae and for 72 h in the region of narrow LPSO lamellae as well as in the matrix in between lamellae with wider distances.

The bar chart in Figure 16 shows the mean values of the multi-indents and the minimum and maximum values of the nanohardness of the as-extruded and the solution heat-treated conditions at 500 °C for 24 h, 48 h, and 72 h. The standard deviation of calculating each individual mean value m_i_ of the multi-indents from the stable range of hardness values, which it was aimed to keep below 0.1 GPa, could not be obtained in the region of LPSO lamellae with narrow distances in the solution heat-treated conditions at 500 °C for 72 h. Although the mean values m_i_ were supposed to be calculated by the hardness values within a range of the lowest dependence on the load applied, in two cases the range of values resulted in a higher standard deviation, 0.2 GPa for lamella 1 and 0.13 GPa for lamella 3 (see Figure 15).

The nanohardness values of the blocky LPSO phases, the matrix with and without LPSO lamellae, of the LPSO lamellae and the matrix in between lamellae with wider distances for 72 h are individually presented in the bar chart in Figure 16. The blocky LPSO phases are, for each condition, harder than the Mg-matrix and their hardness decreases with the solution heat treatment time of 24 h and 48 h and increases slightly at 72 h. The hardness of the Mg-matrix decreases slightly with solution heat treatment for 24 h and then increases with the heat-treatment time for 48 h and further for 72 h. The more detailed investigation of the solution heat-treated condition of 500 °C for 72 h shows a high maximum hardness for LPSO lamellae with narrow distances (3.2 GPa). However, the same region shows a very small hardness values (1.6 GPa), most likely when the lamellae-matrix interface was indented. Therefore, the scattering (the difference between the minimum and maximum values) of the hardness values for these LPSO lamellae regions is the highest. The hardness values of the matrix within LPSO lamellae with wider distances have a rather low scattering (0.11 GPa), with the scattering of the blocky LPSO phases of the as-extruded and the solution heat-treated condition of 500 °C for 24 h (0.02 GPa) and its matrix (0.11 GPa) one of the lowest. The scattering of the hardness values of the blocky LPSO phases in the solution heat-treated condition of 500 °C for 48 h and 72 h (0.5 GPa) is much higher than that of the Mg-matrix conditions (up to 0.3 GPa), but less than the difference in the region of LPSO lamellae in the solution heat-treated condition of 500 °C for 72 h, which, at 1.6 GPa, is very high.

## 4. Discussion

Solution heat treatment at 500 °C clearly causes grain growth. By not considering the blocky LPSO phases due to their higher amount of Dy, Nd, and Zn [25] as small grains, the grain size increases with solution heat treatment at 500 °C with significant scattering. Histograms show the growth of small and medium grains, but some small grains remain within the microstructure. With increasing heat treatment time the number of large grains (around 140 µm) increase. Blocky LPSO phases at grain boundaries will keep the gb mobility down.

The examination of the microstructure by light microscopy has revealed that there are more gb blocky LPSO phases in the solution heat-treated conditions than blocky LPSO phases within the grains. Before solution heat treatment, blocky LPSO phases are found to form gb networks (Figure 3, Figure 7a and Figure 10a), while afterwards there are more gb blocky LPSO than LPSO phases within the grain. The number of blocky LPSO phases decreases with solution heat treatment and with annealing time—especially the number of the inner grain phases. According to the concentration gradient (chemical concentration), the driving force for diffusion of the blocky LPSO phases is expected to be independent of its location. However, when inner grain areas are enriched by stacking faults from hot-extrusion, the diffusion of rare earth elements from blocky LPSO phases into these stacking faults during solution heat treatment is found to form LPSO lamellae [49,50,51,52]. Furthermore, the blocky LPSO phases at the grain boundaries, which are initially preferential nucleation sites [53], are more resistant to dissolution due to their larger size and less pronounced diffusion paths [54]. The level of the interface incoherence will be higher than within the grain, where stacking faults, dislocations and the orientation of atomic planes agree in orientation between the Mg-matrix and inner grain LPSO phase. The level of disruption to the surrounding matrix will be higher for gb LPSO phases, which makes them more stable during solution heat treatment and explains why the value of the ratio between the gb LPSO phases and the inner grains phase increases with annealing time. 

Each of the applied hardness loads HV1, HV0.2, and HV0.005, resulting in different indent sizes or better volumes, provided important and useful information. HV1, with indent sizes of around 160 µm diagonal length, presents hardness values that can be considered as mean values of the heterogeneous microstructure consisting of grains of different size and their grain boundaries and lamellar and block LPSO phases. These HV1 hardness values decrease with solution heat treatment, showing the lowest mean value at 48 h. This hardness decrease during solution heat treatment is a result of fewer LPSO lamellae within a fine-grained microstructure with a significantly smaller amount of blocky LPSO phases and secondary phases [25], but instead, on a more macroscopic level, with coarser grains with less blocky and lamellar LPSO and secondary phases, and therefore lacking gb and precipitation strengthening. It is worth mentioning that the hardness values (although there is a high scattering) show the minimum at 48 h and the fracture toughness shows the highest value at this condition. It seems that the highest energy is needed for crack propagation when a significant amount of blocky LPSO phases have dissolved by using surrounding stacking faults as diffusion paths and Mg-matrix grains became enriched by LPSO lamellae and solid solution, and the grains have not grown too much.

Although the HV0.2 indents are still too large for the fine-grained microstructure as well as for the blocky LPSO phases in the coarse-grained microstructure, the HV0.2 hardness values of the solution heat-treated conditions give evidence that there is a difference in hardness between grains with and without matrix LPSO lamellae, which becomes even more pronounced with the increasing solution heat treatment time. The grains without lamellar LPSO phases, where alloying elements are homogeneously distributed in solid solution, are harder than the grains with LPSO lamellae. LPSO lamellae, as close-packed atomic layers, have Mg-matrix layers of lower hardness in between. By taking a closer look at the microstructure around the indents, imprinted grains without LPSO lamellae consist of more blocky LPSO phases, and the grains are larger than the indent size, so the imprint is not affected by grain boundaries. Furthermore, imprints in grains with lamellar LPSO phases are of larger size than the grain itself, so micro-cracks are inclined to nucleate and propagate at the grain boundaries as well as at lamellar LPSO/Mg-matrix interface, as seen in [32]. This behavior is most pronounced in the heat-treated condition for 48 h, which also supports the highest fracture toughness: sub-cracks form more easily, which extends the crack length and absorbs energy. Grains with LPSO lamellae in the heat-treated condition for 72 h show the smallest mean value of HV0.2; the LPSO lamellae are a wider distance apart, so that the softer Mg-matrix has a greater effect, the grains have grown and fewer grain boundaries are in the measured area and, finally, fewer micro-cracks compared to the heat-treated condition for 48 h are found. The solid solution, by ongoing dissolution of blocky LPSO phases, keeps the hardness value up in grains without LPSO lamellae. The lower scattering in hardness values in the heat-treated condition for 72 h points to the lowest influence of grain boundaries and blocky LPSO phases in the area tested.

The individual HV0.005 values, with diagonal indent lengths of ~10 µm, and especially the nanohardness values, with indent sizes of less than 1.5 µm, can be used to test individual microstructural features. However, due to their lower applied load they cannot be used to discuss and evaluate the hardness values of a higher applied load by their individual hardness values, but by the relation among each other and the general trend. Apart from the HV0.005 indents in blocky LPSO phases, the indent location is free of interfaces between blocky LPSO phases and Mg-matrix as well as grain boundaries. The HV0.005 hardness values of blocky LPSO phases were found to be higher than in Mg-matrix, but their values are influenced by their interfaces: diagonal indent lengths enlarge when hitting their interface to the Mg-matrix, which leads to smaller hardness values. However, the HV0.005 hardness values of the blocky LPSO phases are higher than the other microstructure regions. Agreeing with the HV0.2 hardness values, grains without LPSO lamellae show higher mean hardness values than grains with LPSO lamellae, where the indents were set covering LPSO lamellae and Mg-matrix. When indents were set at selected areas in the Mg-matrix in between the LPSO lamellae (a certain distance was needed for that), the hardness values are the lowest. The softer Mg-matrix consists of fewer solute atoms, which are instead in the LPSO phases in these grains.

The nanohardness values clarify the higher hardness of the blocky LPSO phases: in the extruded condition, blocky LPSO phases reach 160% based on the hardness of the Mg-matrix and, in the solution heat-treated condition at 500 °C, the values exceed 130%. Due to the dissolution process of the blocky LPSO phases, where the phases can also fragment, meaning that their Dy-, Nd- and Zn-enriched layers widen, the decreasing density of rare earth elements in combination with Zn lowers the hardness. On the other hand, the hardness of the Mg-matrix increases with the increasing ageing time from 24 h to 72 h. The Mg-matrix in between the LPSO lamellae shows, in agreement with the HV0.005 hardness values, the lowest hardness. The size of the nanohardness indents even makes it possible to measure the hardness of the LPSO lamellae (or at least very close to them) and a higher mean value compared to LPSO lamellae free of Mg-matrix as well as LPSO lamellae containing matrix can be found. This proves that the Dy-, Nd- and Zn-enriched layers are the hardest features in the microstructure and, in case of indenting them in the right atomic layer, the hardness is even higher than that of the blocky LPSO phases. The significant load dependence of the hardness values of the LPSO lamellae region with narrow distances can be explained by the layered structure. The layers seen in the micrographs will not be cut at an exact 90-degree angle, which means that deeper measured areas will contain more or less Dy-, Nd- and Zn-enriched layers, leading to lower and higher values of one single multi-cycle indent, so-called depth-sensing nanoindentation, which is often found in multilayered coatings or functionally graded materials [55,56].

The evaluation of the hardness of each individual microstructural feature helps to understand and explain the crack propagation and its interaction with the microstructure. Micrographs of the fine-grained microstructure present sub-cracks, which branch off the main crack following the direction of the LPSO lamellae. The Mg-matrix in between the LPSO lamellae is softer and provides the weakest possibility for crack growth. The crack propagation is sidetracked by grain boundaries, due to the different orientation of LPSO lamellae in the neighboring grain, and by blocky LPSO phases due to their higher hardness, where the crack can find its way along the weaker interface of the blocky LPSO phase and Mg-matrix. Since the blocky LPSO phases build a network at the gb, the weaker interfaces have been found to carry the crack from grain to grain.

The grain growth during solution heat-treated conditions at 500 °C allows twinning during plastic deformation. These twins also interact with the crack propagation: the crack surface appears jagged and uneven; a zig-zag crack pattern is the result of the crack following the twin boundaries, as seen for LPSO lamellae. These twin boundaries cause an accumulation of dislocation on new slip traces, finally leading to regions of secondary micro-cracking [57]. Slip-controlled crack growth is also reported in [58], leading to a main crack, which follows a zig-zag path controlled by the development of slip traces near its tip. Any extension of the crack length increases the fracture energy.

The knowledge about the nanohardness helps to understand why the crack follows the lamellar LPSO phases within the Mg-matrix: due to a larger distance between the LPSO lamellae, a higher amount of dislocation gathers in the softer Mg-matrix at the interface of the LPSO lamellae, and micro-cracks initiate the crack propagation along the lamellae. The sub-cracks interact with LPSO lamellae, which disagree with the growth direction, and the LPSO lamellae redirect the crack in their own direction, until the main loading direction, given by the geometrical specimen shape and size during C-ring compression, will force it towards the next LPSO lamellae, also leading to a zig-zag pattern.

The micrographs studied show that some blocky LPSO are crossed and some are passed sideways. Crossing the blocky LPSO phases is possible when the orientation of the lamellar structure agrees with the crack propagation or is not far off it. However, according to the higher hardness of the blocky LPSO phases, stresses pile up and more energy will be needed. Disagreement leads either to crack blunting or passing the blocky LPSO phase sideways along the interface [59]. Any more energy needed for that increases the fracture toughness. The microstructure that develops during solution heat treatment at 500 °C for 48 h consists of blocky LPSO phases at the grain boundaries, and lamellar and blocky LPSO phases to redirect grain growth, but of a limited amount, allowing softer matrix in between to form a plastic deformation zone within the coarser grains, which are not too large, and this microstructure seems to absorb the highest energy. Due to its higher hardness, the blocky LPSO phase will have increased brittleness. That is why cracked blocky LPSO phases have been found to initiate cracks. When coarse grains are free of twins, lamellar and blocky LPSO phases, the crack runs continuously to the next gb, where the energy needed is influenced by the amount of solute atoms (solid solution strengthening).

This study clearly helps to understand the role of blocky and lamellar LPSO phases on crack propagation and, thus, on the fracture toughness. The blocky LPSO phases are of a certain high hardness, providing some strength in general, but also serving as obstructions to crack growth; inner grain blocky LPSO phases are here very effective. In the fine-grained microstructure, the crack, which mainly follows the LPSO lamellae, can easier carry on growing along the LPSO lamellae in the neighboring grains, instead of being hindered by perpendicular lamellas in other grains. Current research is focusing on the detailed explanation of the high fracture toughness in the solution heat-treated condition at 500 °C for 48 h, so more nanohardness data are to come.

## 5. Conclusions

The effect of blocky and lamellar LPSO phases and their hardness on the crack propagation in different microstructures of Mg-Dy-Nd-Zn-Zr alloy (RESOLOY) has been investigated. The fine-grained microstructure consists of lamellar LPSO structures within the matrix and in the coarser grains there are fewer lamellae, but blocky LPSO phases, whose amount decreases with heat treatment time, especially the inner grain blocky phases. The amount and location of LPSO phases were evaluated by optical microscopy.

C-ring compression tests in Ringer solution were done to cause sample failure. Crack initiation and propagation were demonstrated with the focus on the interaction of the microstructural features and the crack growth. Twins and LPSO lamellae are responsible for crack initiation. The blocky LPSO phases clearly hinder crack growth, by increasing the energy needed to pass either through the phase or along its interface. When the crack growth direction is off the orientation of the matrix LPSO lamellae, they also redirect the crack. The crack runs in the softer Mg-matrix in between the LPSO lamellae. This softer Mg-matrix layer provides the possibility for slip band cracking. In the coarser-grained microstructure, the crack propagation is also influenced by twin boundaries, especially on the compression side of the C-ring sample.

The microstructure, with a focus on microstructural features, was characterized by micro- and nanohardness. Hardness testing with a lower load was needed to measure the hardness of the blocky LPSO phases and LPSO lamellae individually (HV0.005, and much more specifically, nanohardness). Blocky LPSO phases show a higher hardness than the grains with or without lamellar LPSO phases. The matrix in between matrix LPSO lamellae shows the smallest values. Whereas the blocky LPSO phases decrease with heat treatment time, the grains free of lamellar LPSO phases increase in hardness. The dissolution of blocky LPSO phases enriches the matrix by solid solution.

The highest hardness and strength was found in the fine-grained extruded condition with grains completely filled by lamellar LPSO phases. However, the highest fracture toughness is seen in the solution heat-treated condition at 500 °C for 48 h, where the hardness shows the lowest value and the microstructure has a good mix of grain sizes, lamellar and blocky LPSO and twins formed under the mechanical loading. This study shows that the properties of the alloy system Mg-Dy-Nd-Zn-Zr can be easily tailored by post heat treatment. Since other studies have provided good corrosion resistance, it makes this alloy system an interesting candidate among the Mg-RE alloys.

## Data Availability

Not applicable.
